# Inconsistent and Misleading Expressions

**Published:** 1902-07

**Authors:** B. F. Arrington

**Affiliations:** Goldsboro, N. C.


### INCONSISTENT AND MISLEADING EXPRESSIONS.

BY B. F. ARRINGTON, GOLDSBORO, N. C.

Some men of prominent distinction in the dental profession sometimes give utterance to expressions pertaining to theory and to practical matters in dental practice that are very inconsistent, entirely out of line of all reason, and hurtfully misleading, especially with that class of dentists who accept statements and teaching from what they regard high sources, without ever questioning whether they practically be reasonable or unreasonable, true or false. There are such dentists, many of them.

We are all liable to be misled, and are liable to err in our practice and in statements concerning practice, but some err to injury to a greater extent than others. The more prominent and professionally conspicuous the man or men making unreasonable and inconsistent statements, such as will not admit of favorable sanction when put to the test, the more hurtful and the greater the injury done, therefore the necessity and demand for thoughtful consideration and careful investigation of all seemingly extreme, unreasonable, and impractical statements emanating from such sources. It is the impractical in practice that retards true progress.

We will for the present refer to but one subject for consideration, and will be brief in pointing to some statements made by a distinguished member of the profession concerning the manipulation of amalgam and the results. Dr. Black is too favorably established in the esteem and confidence of the profession to be unfavorably affected by just and fair criticism, consequently I do not hesitate to criticise as cause justifies, that some of the profession, if not profited, may be induced to pause and reflect before too readily accepting as correct and valid theory and practice that cannot and will not hold good when subjected to legitimate tests. He is quoted prominently in dental journals, and is at present generally regarded, possibly, as our highest authority pertaining to dental alloy and amalgam practice. As reported in one journal, he said to a dozen dentists, "Make the fillings from the same bottle of alloy just as you please, use much mercury or little mercury, knead it much or little as you think proper; use much or little pressure in packing the filling as you please, so you use such as would be used in the mouth; simply, I do not expect you to put seven or eight hundred pounds on it in packing. Now, every one of that dozen fillings will shrink or expand practically alike." In another journal he says, "I should expect that severe grinding amalgam in a wedgewood or roughened glass mortar would considerably increase the contraction." He says he "uses steady, down pressure, and fills with flat surface, serrated pluggers, using comparatively light pressure." In another journal we find him saying, "We need more pressure in condensing amalgam than in condensing gold." He also says, "When the minimum pressure for condensing gold is fifteen pounds he wants twenty pounds for amalgam."

Here are statements seemingly inconsistent, and are unquestionably misleading. Strictly accepting and following such inconsistent and unreasonable theory and practice, who could be expected to fill creditably and give satisfaction in the use of amalgam?

Such teaching and practice, should it become general, would preclude the possibility of best results in the use of amalgam.

Progressive practice up to date, on a reasonable and sound basis, is what we want and must have. If in our efforts so far to accomplish desired results we have failed, we must persevere until success is accomplished.

In teaching, a consistent, practical line of practice pertaining to use of material for the preservation of teeth is to be respected and followed for success and a guarantee of satisfactory results.

To teach theory and practice that is not in conformity with sound judgment and common-sense reasoning, and will not stand fair tests, is unreliable and hurtful in results, especially so in our treatment and care of the natural teeth.

For true advancement and the accomplishment of work that will stand proper tests and prove theory and practice correct, we must, when we realize that we have erred, amend our teaching and practice, and work energetically and faithfully to get on line and in line, if possible, of correct theory and conservative, practical practice, for that line alone is reliable and will stand the test of time, and securely hold the stamp of truth. Our period for life-work is too limited to admit of retracing, undoing, and redoing, if to be avoided.

